# IL-4R alpha deficiency influences hippocampal-BDNF signaling pathway to impair reference memory

**DOI:** 10.1038/s41598-020-73574-3

**Published:** 2020-10-05

**Authors:** Tiroyaone Masego H Bogale, I. Berkiks, S. Pillay, M. Scibiorek, B. O. Moses, F. Brombacher

**Affiliations:** 1 International Centre for Genetic Engineering and Biotechnology (ICGEB), Cape Town Component, Cape Town, South Africa; 2 Division of Immunology, Institute of Infectious Disease and Molecular Medicine (IDM), Health Science Faculty, University of Cape Town, Wernher and Beit Building, South, Cape Town, 7925 South Africa

**Keywords:** Behavioural methods, Immunological techniques, Immunology, Molecular biology, Neuroscience, Psychology

## Abstract

Like pro-inflammatory cytokines, the role of anti-inflammatory cytokines in both learning and memory has been investigated, revealing beneficial effects for both interleukin-4 and interleukin-13 via the common interleukin-4 receptor alpha chain complex. In this study, using the Morris water maze spatial task for cognition, we compared interleukin-4 receptor alpha- deficient mice and their ligands interleukin-4/ interleukin-13 double deficient mice, on a Balb/c background. We demonstrate that while interleukin-4/ interleukin-13 double deficient mice are significantly impaired in both learning and reference memory, interleukin-4 receptor alpha-deficiency impairs only reference memory, compared to the wild-type control mice. In order to better understand how interleukin-4 receptor alpha- deficient mice are able to learn but not remember, we investigated the BDNF/TrkB- and the ARC-signaling pathways. We show that interleukin-4 receptor alpha-deficiency disrupts activation of BDNF/TrkB- and ARC-signaling pathways during reference memory, while the pathway for spatial learning is spared.

## Introduction

The immune and central nervous systems (CNS) are capable of influencing healthy functioning of the brain^1^, with cytokines interleukin-4 (IL-4) and interleukin-13 (IL-13) reported to affect both learning and reference memory^2,3^ by influencing brain derived neurotrophic factor (BDNF) production^2,4,5^. IL-4 signaling effects are mediated via IL-4 receptor alpha (IL-4Rα) chain, where upon binding to ligands IL-4Rα dimerizes with either common gamma chain (γc) for type-1 signaling, or with IL-13 receptor alpha 1 (IL-13Rα1) for type-2 signaling^6–9^. IL-13 mediates its effects by binding to either IL-13Rα1 or IL-13Rα2 for type-2 signaling^10,11^.

BDNF receptor tropomyosin-related kinase B (TrkB) and activity-regulated cytoskeleton-associated protein (ARC) are known for their interactive importance for spatial memory in the hippocampus^12–15^. Transcription factor c-AMP-responsive element binding protein (CREB) regulates gene expression for both adaptive neuronal responses^16^, including BDNF, and complex functions that involve learning and memory^17,18^. Basically, downregulation of CREB expression is associated with dementia, while increased expression is considered as a likely therapeutic target for memory-loss disorders^19^.

IL-4 and IL-13 have been shown to play significant roles in brain and spinal cord repair by regulating microglia and macrophages function^20^. IL-4 deficiency leads to sensory motor deficits and in turn impaired cognitive function, however signaling via IL-4Ra is essential^21^. While decrease in IL-4Ra positive macrophages recruitment to a site of injury or infection has been linked to inability to promote IL-4 signaling via IL-4Ra, impairment in induction of IL-4Ra on microglia is noted as a contributing factor to reduced recovery^22^.

Because IL-4 and IL-13 share the IL-4Rα-chain to support spatial learning and reference memory^8,10^, we investigate whether double deletion of IL-4 and IL-13 would yield similar spatial cognitive results as deleting their shared receptor IL-4Rα. We aimed to probe involvement of BDNF and its receptor TrkB in reference memory formation pathway that engages transcription factor CREB and ARC in the hippocampus. These results may be important for future development of therapeutic approaches associated with neurocognitive disorders linked to long-term memory loss and dementia.

## Results

### Deletion of IL-4Rα does not yield similar behavioral results as double-deletion of cytokines IL-4 and IL-13

**Figure 1:**
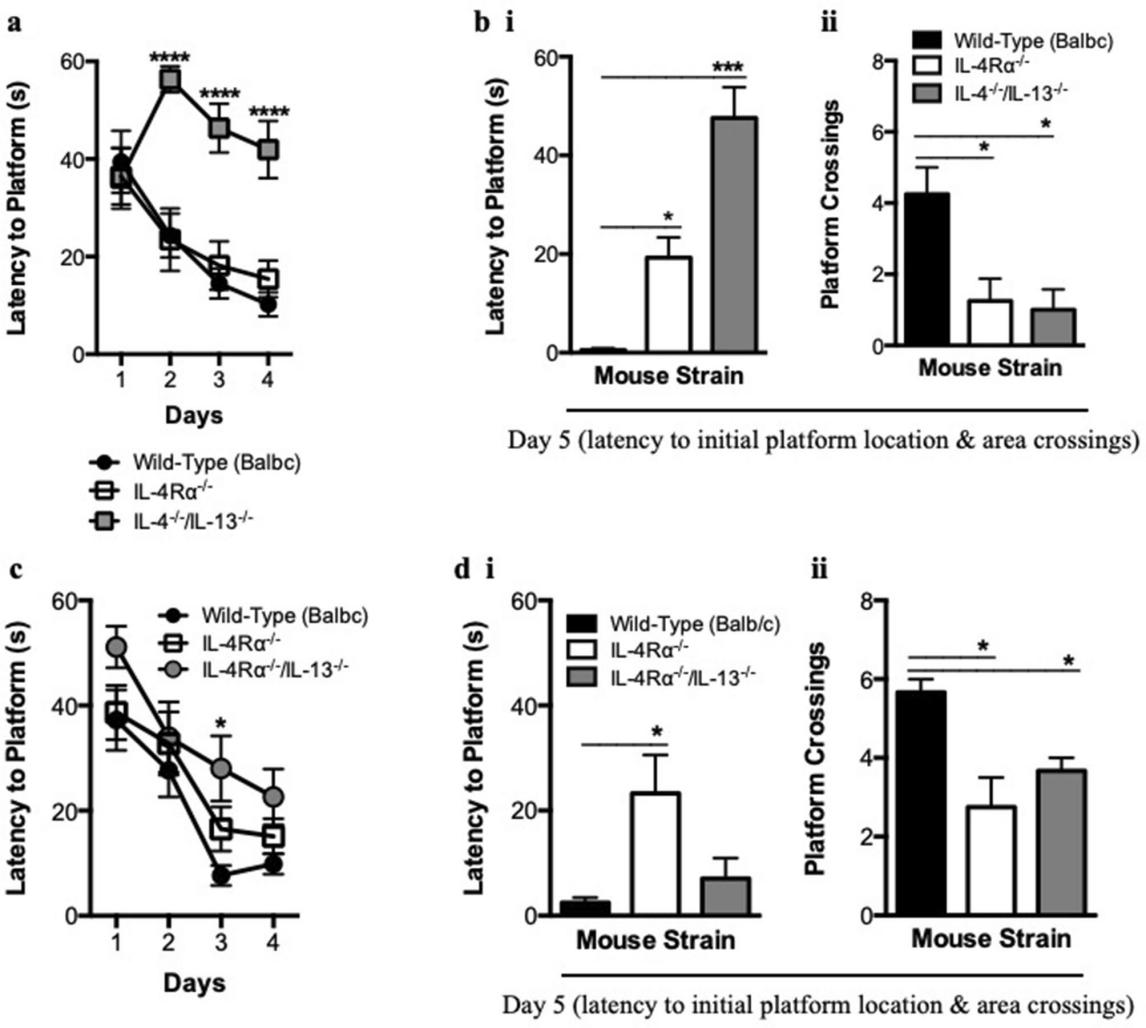
Loss-of-function cytokine and receptor induced cognitive defects. Male mice (8 wks old) were investigated for learning and reference memory in the MWM. (**a**) Compared to wild-type mice, IL-4Rα^−/−^ mice showed similar decreasing latencies to platform location during the acquisition phase of the task, while IL-4^−/−^/IL13^−/−^ mice had longer latencies. (**b**, **i**) IL-4Rα^−/−^ and IL-4^−/−^/IL13^−/−^ mice demonstrated longer latencies to platform location, (**b**, **ii**) with fewer platform crossings compared to the wild-type control mice. (**c**) Compared to IL-4Rα^−/−^ mice, IL-4Rα^−/−^/IL-13^−/−^ mice showed similar decreasing latencies to platform location during the acquisition phase of the task. (**d**, **i**) IL-4Rα^−/−^ mice demonstrated significantly longer latencies to platform location, (**d**, **ii**) while both IL-4Rα^−/−^ and IL-4Rα^−/−^/IL13^−/−^ mice had fewer platform crossings compared to wild-type control mice (n = 6 mice per group; 2-way repeated measures ANOVA with Bonferroni post hoc test; unpaired t test, *P < 0.05; ***P < 0.001; ****P < 0.0001). Results are representative of five independent experiments.

IL-4Rα, like cytokines IL-4 and IL-13, could be essential for cognitive function. This is investigated in a loss of function approach using IL-4Rα- and IL-4/IL-13-double deficient mice for spatial cognition^23^. Wild-type, IL-4^−/−^/IL-13^−/−^, and IL-4Rα^−/−^ Balb/c mice were trained in the MWM, where wild-type and IL-4Rα-deficient mice showed similar latencies to platform compared to IL-4/IL-13-double deficient mice (Fig. 1a). Interestingly, although IL-4Rα deletion led to unimpaired learning compared to the wild-type control, this receptor deficiency like double deletion of IL-4/IL-13 shows longer latencies to platform location, with fewer platform crossings compared to wild-type control (Fig. 1b).

### IL-13 does not signal via IL-13Rα2 in the absence of IL-4Rα to support cognition

In an attempt to better understand cognitive effects associated with IL-4Rα deletion, the possibility of IL-13 signaling via IL-13Rα2 to support learning but not reference memory was investigated. Wild-type, IL-4Rα^−/−^ and IL-4Rα^−/−^/IL-13^−/−^ Balb/c mice were tested in the MWM task for both learning and reference memory, showing similar latencies to platform for all three groups (Fig. 1c). However, impaired reference memory was noted for IL-4Rα- and IL-4Rα/IL-13-double deficient mice compared to wild-type control mice, characterized by longer latencies to platform location and fewer platform crossings (Fig. 1d). It is evident when taking both latency to platform location and number of platform area crossings into consideration in order to determine successful spatial reference memory formation, that both IL-4Rα- and IL-4Rα/IL-13-double deficient mice are consistent in not being statistically different from each other. This result suggests that IL-4Rα-deficiency impacts on reference memory independent of IL-13, despite IL-4Rα/IL-13-double deficient mice showing seemingly shorter latencies to platform location.

### IL-4Rα activity determines MWM spatial task reference memory by supporting TrkB receptor- and ARC-expression

**Figure 2:**
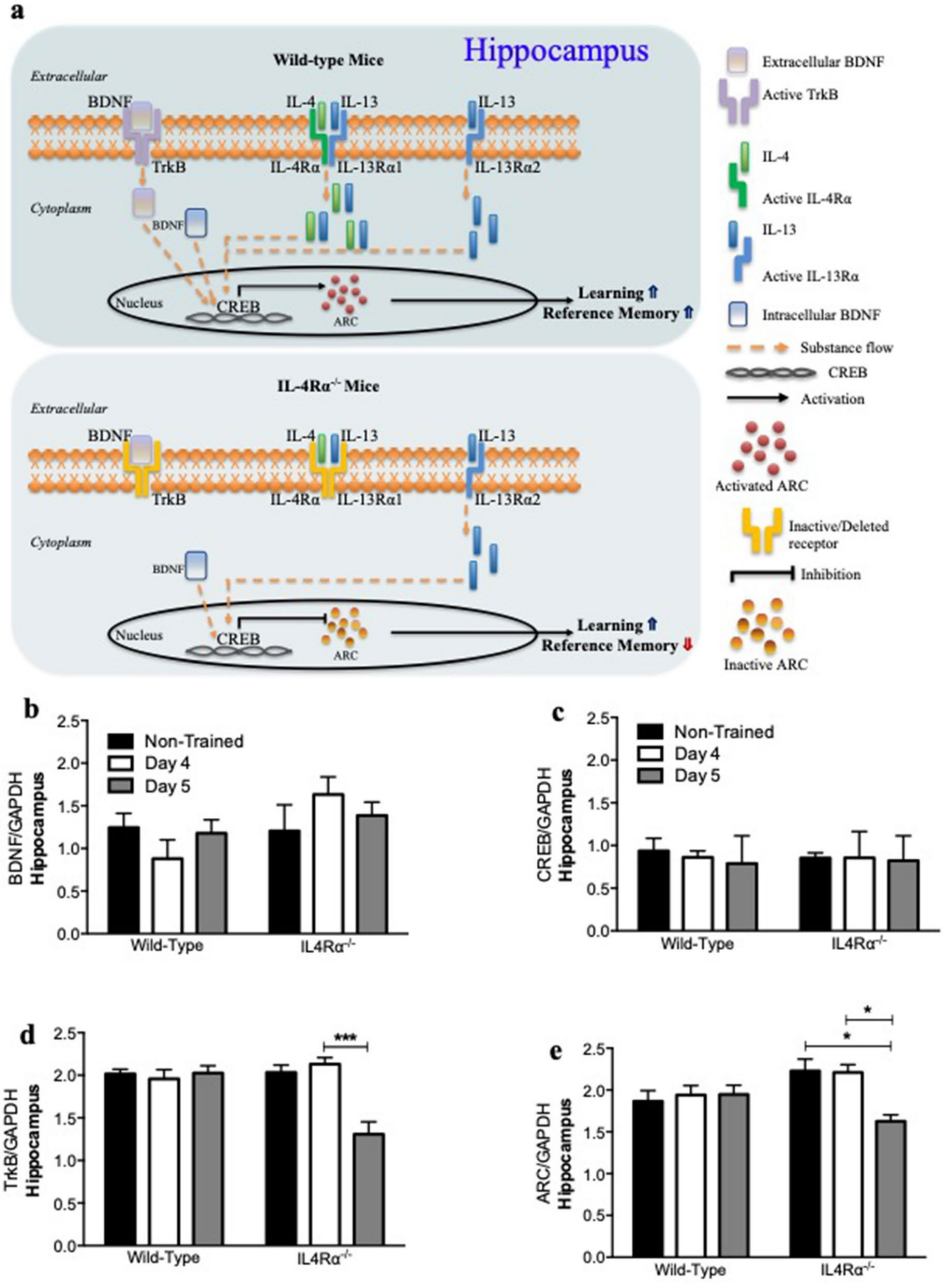
BDNF signaling pathway in relation to IL-4Rα deficiency during spatial task. (**a**) A schematic representation of day 4 (acquisition) and day 5 (reference memory) in the hippocampus is shown for TrkB receptor responses in the Morris water maze spatial task to support learning and reference memory. From single cell suspensions, by means of qPCR, CREB, BDNF, TrkB and ARC production were determined following spatial task at day 4 and day 5, compared to non-trained mice. There were no strain differences shown for (**b**) BDNF and (**c**) CREB expression across training days. However, in the hippocampus, compared to non-trained and day 4, IL-4Rα^−/−^ mice show significantly reduced (**d**) TrkB receptor and (**e**) ARC expression on day 5 (n = 4/5 mice per group; 2-way ANOVA with Bonferroni post hoc test, *P < 0.05; ***P < 0.001). Results are representative of two independent experiments.

Having determined that effective learning by IL-4Rα-deficient mice is not due to IL-13 signaling via IL-13Rα2, we investigated the possible role of BDNF signaling pathways during learning and reference memory phases of the task for wild-type and IL-4Rα^−/−^ mice. Results show steady expression levels of BDNF and CREB in the hippocampus (Fig. 2a–c), that correlate directly to successful learning for wild-type and IL-4Rα-deficient mice (Fig. 1a). Furthermore, during the reference memory phase of the task (day 5), the effects of steady expression levels of BDNF and CREB observed were disrupted by attenuated TrkB receptor- and ARC-expression in the hippocampus (Fig. 2d,e) that correlates to impaired reference memory (Fig. 1b).

### Increased myeloid cell populations support learning of IL-4Rα-deficient mice

**Figure 3:**
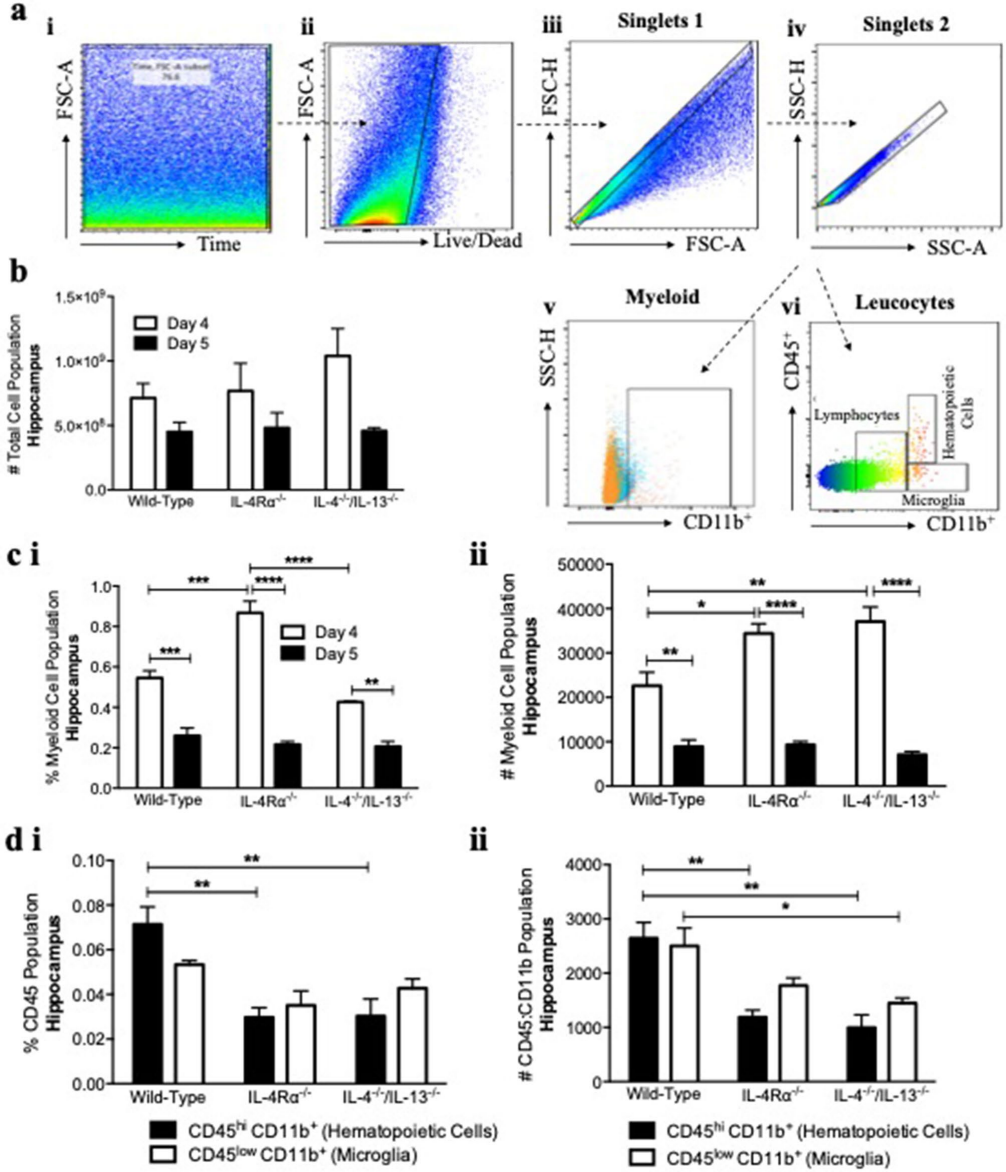
Myeloid cell involvement in reference memory of IL-4Rα^−/−^ mice. By means of flow cytometry, single-cell suspensions from the hippocampus were examined for CD45+ CD11b+ myeloid cells. (**a**) A gating strategy for CD45^hi^CD11b+ and CD45^low^CD11b+ myeloid cell population is shown. (**b**) Total cell numbers were determined showing no differences across strains on both days. (**c**, **i**, **ii**) More myeloid cells by events and numbers on day 4 (acquisition) compared to day 5 (reference memory) are shown across strains, with (**c**, **i**) high events of myeloid cells shown for IL-4Rα^−/−^ mice compared to wild-type and IL-4^−/−^/IL13^−/−^ mice on day 4. (**d**) On day 5, wild-type mice show significantly more CD45^hi^CD11b+ (hematopoietic cells) events and numbers than IL-4Rα^−/−^ mice and IL-4^−/−^/IL13^−/−^ mice, with significantly more CD45^low^CD11b+ (microglia) numbers than IL-4^−/−^/IL13^−/−^ mice (n = 5 mice per group; unpaired t test, **P < 0.01; ***P < 0.001; ****P < 0.0001). Results are representative of two independent experiments.

Having shown that IL-4Rα- and IL-4/13-double deficiency leads to impaired reference memory by means of dampened TrkB receptor- and ARC-expression, we probed into the role of myeloid cells on day 5 for a measure of reference memory. A gating strategy to identify CD11b^+^ cell population is shown (Fig. 3a) with results revealing similar total numbers across strains for day 4 and day 5 on a measure of learning and reference memory (Fig. 3b). While there are no myeloid cell population differences on day 5 across strains, IL-4Rα-deficient mice show significantly more cells on day 4 compared to wild-type and IL-4/13-double deficient mice, possibly aiding to support learning to be similar to wild-type mice. There are also consistent higher amounts of myeloid cells on day 4 compared to day 5 across strains (Fig. 3c).

### Reduction in hematopoietic cells for IL-4Rα- and IL-4/13-double deficient mice contributes to impaired reference memory

To determine possible roles of CD45^hi^CD11b^+^ (hematopoietic cells) and CD45^low^CD11b^+^ (microglia), these populations were investigated^24–26^ revealing significantly lower hematopoietic cells (Fig. 3d i, ii) and microglia (Fig. 3d ii) for IL-4Rα- and IL-4/13-double deficient mice compared to wild-type controls on day 5. This result correlates to impaired reference memory by IL-4Rα- and IL-4/13-double deficient mice.

## Discussion

The hippocampus is an area of the brain implicated in long-term potentiation of spatial learning tasks^12,27,28^. We show that although IL-4Rα-deficient mice learn the MWM task equally well as wild-type mice (Fig. 1a), they demonstrate significant impairment for reference memory (Fig. 1b). In this study we provide evidence for the requirement of BDNF/TrkB/CREB/ARC pathway in partnership with cytokines IL-4 and IL-13 signaling via IL-4Rα for successful learning and reference memory. Moreover, while an increase in myeloid cells on day 4 appear to support learning for IL-4Rα-deficient mice, reduced hematopoietic cells and microglia cell numbers during the probe trial contribute to impaired reference memory.

We unveil that IL-4/IL-13-double deficiency leads to impaired learning; a result that confirms published data implicating the importance of cytokines IL-4 and IL-13 in spatial learning^2^. A surprising result is that of IL-4Rα-deficiency yielding similar results of effective learning as wild-type mice, contrary to an expected result of IL-4Rα-deficiency leading to impaired learning, similar to IL-4 and IL-13 deficiency. To further investigate how IL-4Rα-deficient mice are capable of learning similar to wild-type mice, we probed into the possibility of IL-13 supporting successful learning by signaling via an alternate receptor IL-13Rα2. Using a loss-of-function approach, mice were tested on a task of learning using a strain deficient of both IL-4Rα and IL-13. This ensured that IL-4 could not signal via IL-4Rα, while there was no IL-13 to signal via either IL-4Rα or IL-13Rα2. The IL-4Rα/IL-13-double deficient mice showed a successful learning result similar to wild-type and IL-4Rα-deficient mice, suggesting that the ability of IL-4Rα-deficient mice to successfully learn the Morris water maze (MWM) spatial task may not be attributed to IL-13 signaling via IL-13Rα2, but rather to an alternate pathway.

Although IL-4Rα-deficient mice are capable of learning the MWM spatial task equally well as wild-type mice, they display impaired reference memory. We aimed to investigate a signaling pathway that does not rely on IL-4 and IL-13 signaling via IL-4Rα to support learning, while requiring this activity for effective reference memory. From a molecular approach, this reference memory impairment by IL-4Rα-deficient mice can be directly linked to attenuated TrkB receptor- and ARC-expression^12–15^ (Fig. 2d, e), along with reduced hematopoietic cells and microglia populations (Fig. 3d). Basically, what this result in the hippocampus suggests is that during the probe trial when the platform is removed from its initial location and mice are expected to remember where it was placed, IL-13 that signals via IL-13Rα2 along with intracellular BDNF support CREB activity in IL-4Rα-deficient mice. Nevertheless, without cytokines IL-4 and IL-13 signaling via IL-4Rα^29^, along with no extracellular BDNF signaling via TrkB receptor, these mice fail to initiate ARC expression, in turn leading to impaired reference memory during the probe trial. Furthermore, IL-4Rα-deficient mice demonstrate lower counts of hematopoietic cells and microglia compared to wild-type mice that could very well be contributors to the reference memory impairment observed, by a mechanism yet to be investigated.

In conclusion, while double deletion of cytokines IL-4 and IL-13 impairs both learning and reference memory, IL-4Rα deletion impairs reference memory, but not learning. This IL-4Rα deletion result of impaired reference memory is attributed to failure of extracellular BDNF to signal via TrkB receptor in collaboration with cytokines IL-4 and IL-13 that are unable to signal via IL-4Rα, enabling only successful learning. We attribute wild-type mice successful learning and reference memory to extracellular BDNF that binds to TrkB receptor allowing for both extracellular and intracellular BDNF to collaborate with the IL-4 and IL-13 that signal via IL-4Rα to enable transcription factor CREB to activate ARC (Fig. 2a). IL-4Rα-deficient mice rely on IL-13 that signals via an alternate receptor IL-13Rα2 to collaborate with only intracellular BDNF that fails to enable transcription factor CREB to activate ARC, leading to successful learning, but impaired reference memory (Fig. 2a). This is based on loss of function approach suggesting that in the absence of IL-4Rα/IL-13Rα1 heterodimer (receptor subunit for IL-4 and IL-13), IL-13 can only exert its effects on cognitive function by signaling via the only other available receptor subunit, which is IL-13Rα2^30,31^.

## Materials and methods

### Animals

Inbred 8-wk-old IL-4Rα-deficient^32^, IL-4 and IL-13-double deficient^33^, IL-4Rα and IL-13-double deficient generated in our laboratory using a cre/lox method, as well as wild-type littermate control mice of Balb/c genetic background were obtained from the University of Cape Town specific pathogen-free animal facility and kept in individually ventilated cages. All animals were housed in temperature- and humidity-controlled rooms, maintained on a 12 h light/dark cycle and age matched in each experiment. Animal protocols were approved by the independent Animal Ethics Research Committee at the University of Cape Town (approval no. 015/050), and all methods were performed in accordance with the relevant guidelines and regulations.

### Morris water maze

Spatial learning and reference memory function of mice were investigated in the MWM for five days. During acquisition, mice were given four 5-min trials a day for 4 days to locate a submerged plexiglass circular platform (10 cm in diameter), that was removed on day 5 to test for reference memory as previously detailed^2^. Data were recorded using the EthoVision XT 8 automated tracking system (Noldus Information Technology, Leesburg, VA), and statistical analyses performed using Student *t* test, or analysis of variance (ANOVA), with a Bonferroni post hoc test. Groups were run in alternating order on successive training days, with all MWM experiments conducted between 9:00 am and 3:00 pm during the lights-on phase. Shown are representative experiments out of a minimum of at least three independently performed in each case.

### Brain sample collection

On either day 4 or day 5 post MWM training, mice were euthanized with halothane to collect brain tissue samples. The hippocampus was isolated according to a modified protocol following transcardial perfusions^34^ and collected into CentriStar cap 15 ml Corning centrifuge tubes (Corning, NY) in Isove’s Modified Dulbecco’s Medium (IMDM) (GIBCO/Invitrogen; Carlsbad, CA), 10% Fetal Calf Serum (FCS), and penicillin streptomycin (P/S) on ice. Tissue was pushed through 40 μm nylon cell strainers (Falcon, Corning Incorporated, NY) and centrifuged at 1200 rpm at 4 °C for 10 min to be used for flow cytometry. Other samples were snap frozen for qPCR.

### Quantitative RT-PCR

#### RNA extraction

Isolated brain tissue samples suspended in *QIAzol* Lysis Reagent were homogenized in beads at 4 °C using TissueLyser LT, followed by total RNA extraction using RNeasy Mini kit, according to manufacturer’s instruction (Qiagen, Switzerland). RNA purity and quantification were determined by NanoDrop ND-1000 Spectrophotometer (NanoDrop Technologies, Delaware, USA).

#### cDNA synthesis and RT-qPCR

cDNA was synthesized from RNA extractions, that were reverse-transcribed using Transcriptor First Strand cDNA Synthesis Kit (Roche, Germany), and gene expression determined by quantitative real-time PCR (RT-qPCR) using LightCycler 480 SYBR Green I Master (Roche, Germany). The fold change in gene expression was calculated by the ΔCt method and normalized to GAPDH as the internal control. Primers used in the study are as shown in Table 1:

**Table 1:** Primers used in the study.

Gene names	Primer sequences
BDNF	Forward: 5′CGGTACAGTTGGCCTTTGGATACCG-3′ Reverse: 5′GTGGGTCACAGCGGCAGATA-3′
CREB	Forward: 5′ACTGGCTTGGCACAACCAGA-3′ Reverse: 5′GGCAGAAGTCTCTTCATGATT-3′
ARC	Forward: 5′CTCCAGGGTCTCCCTAGTCC-3′ Reverse: 5′TGAGACCAGTTCCACTGCTG-3′
TrkB	Forward: 5′TGACGCAGTCGCAGATGCTG-3′ Reverse: 5′TTTCCTGTACATGATGCTCTCTGG-3′
GAPDH	Forward: 5′TTCACCACCATGGAGAAGGC-3′ Reverse: 5′GGCATGGACTGTGGTCATGA-3′

#### Flow cytometry

Single cell suspensions were stained with an Ab mix (MACS buffer plus 2% inactivated rat serum), 2% anti-FcγII/III (clone 2.4G2), anti-CD11b (clone M1/70; BD Horizon), and anti-CD45 (clone 30-F11; BD Pharmingen) for 45 min on ice, and then fixed in 2% paraformaldehyde. Samples were read using a BD FACS Fortessa machine (BD Biosciences, San Diego, CA), and data analysed by FlowJo (Tree Star, Ashland, OR) to be graphed with GraphPad Prism software.

### Statistical significance

Statistical significance was measured by two-tailed unpaired student *t* tests, or ANOVA corrected for multiple comparisons with a Bonferroni post-hoc. GraphPad Prism v 6.0 was used for analyses, with a ‘*p*’ value of less than 0.05 considered significant (*p < 0.05, **p < 0.01, ***p < 0.001, ****p < 0.0001).

## Abbreviations

IL-4: Interleukin-4
IL-13: Interleukin-13
IL-4Rα: Interleukin-4 receptor alpha
BDNF: Brain-derived neurotrophic factor
CREB: C-AMP-responsive element binding protein
TrkB: Tropomyosin-related kinase B
ARC: Activity-regulated cytoskeleton-associated protein
MWM: Morris water maze
Balb/c: Bagg albino
γc: Gamma chain
CNS: Central nervous system
